# Self-Consistent Field Analysis of Segregative Aqueous Dextran–Polyethylene Glycol Solutions: (2) Adsorption and Wetting

**DOI:** 10.1021/acs.jpcb.5c04279

**Published:** 2025-10-14

**Authors:** F. A. M. Leermakers, L. Ruiz-Martínez, S. D. Stoyanov, J. van der Gucht

**Affiliations:** † Physical Chemistry and Soft Matter, Wageningen University, Stippeneng 4, Wageningen 6708 WE, The Netherlands; ‡ Food, Chemical, and Biotechnology Cluster, Singapore Institute of Technology, 10 DoverDrive, Singapore 138683, Singapore

## Abstract

Aqueous two-phase systems (ATPS), with dextran-water-poly­(ethylene glycol) (PEG) as the main example, are water-continuous polymer segregated systems. How such ATPS behaves near solid interfaces is studied using the Scheutjens Fleer self-consistent field (SF-SCF) theory. We have analyzed the adsorption isotherms of the minority (wetting) component PEG at either a fixed solvent- or a fixed dextran bulk volume fraction and focused on wetting and polymer displacement transitions. When the driving force for segregative phase behavior is sufficiently strong (repulsive interactions between dextran and PEG: the major driving force), a jump-like transition displacing adsorbed dextran by PEG may take the form of a prewetting transition when the PEG-rich phase wets the surface. Interestingly, the first-order displacement transition manifests as a pure surface phase transition at supercritical conditions. This explains why the wetting transition is robust first order. Only when the segregative phase behavior is triggered by a solvent quality disparity (minor driving force), one may find isotherms with two consecutive transitions, that is, a smooth displacement transition followed by a jump-like prewetting transition. Explained by the low interfacial tension between the PEG-rich and dextran-rich phases, the parameter window for partial wetting is small. Remarkably, for an experimentally realistic ATPS and carefully tuned adsorption parameters, it is possible that upon a change of the solvent content there is a sequence of two wetting transitions (going from partial wet to wet and back to partial wet) followed by a drying transition.

## Graphical abstract

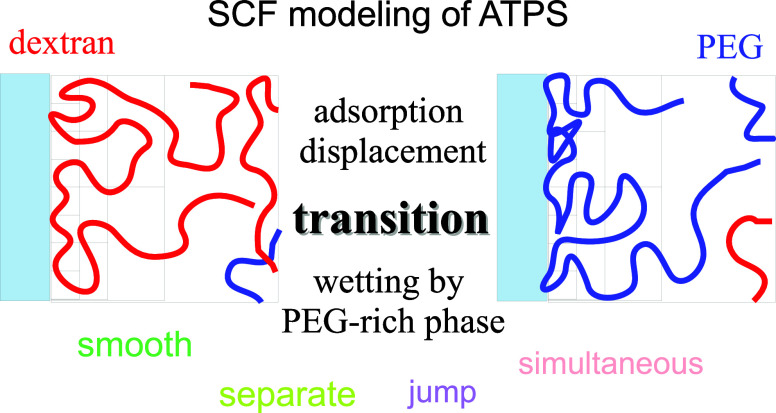

## 1 Introduction

When colloidal particles that readily disperse in water, such as silica (SiO_2_), hematite (Fe_2_O_3_) or poly­(styrenesulfonate) (PSS), are added to an aqueous two phase system (ATPS) such as dextran-water-Poly­(ethylene glycol) (dextran-water-PEG), two scenarios exist:^1–3^ (i) the particles are interfacially active, and one may form water in water Pickering emulsions^4^ (ii) the particles do not adsorb at the interface and either disperse in the PEG-rich or in the dextran-rich phase. In the latter scenario, one may generate capillary suspensions,^5,6^ in particular when the phase preferred by the particles is the minority phase and the particle concentration is sufficiently high. Facilitated by the wetting preference, the minority phase forms capillary bridges^7^ between particles that, by passing the percolation threshold,^8^ form an elastic network. Macroscopically, this is clearly noticed as the capillary suspension no longer flows as a fluid, but behaves like a gel that can withstand gravitational forces.^5^

Both scenario’s are of interest for applications. When particles are interfacially active, an oil-free, low-calorie Pickering emulsion can penetrate the food market when edible compounds such as gelatin, particles composed of protein molecules, and the like are used.^3,9^ Alternatively, capillary suspensions find applications in products that require a yield stress and a high modulus, for example, in the production of porous materials.^10^

In the above, it was suggested that a wetting transition separates these two types of behavior. However, this is not strictly the case.^5,11^ For example, provided that one carefully selects the correct conditions, that is, high particle loading and the phase preferred by the particles is truly the minority phase, it is possible to have capillary suspensions even when the particles are still interfacial active, meaning that the contact angle is less than 90°. However, applications benefit from low contact angles. Clearly, some understanding of the wetting characteristics in these systems is important to rationalize the properties of capillary suspensions and to make designer choices.^12^

In an ATPS, by definition, we have a system with a solubility gap. We have shown before that there are two possible driving forces that lead to segregative phase behavior.^13^ (i) The first option is that the two polymer components repel each other (main driving force).^14^ In this case, a PEG-rich phase and dextran-rich phase forms, while both phases remain rich in solvent (water). In a phase diagram, typically the coexisting polymer concentrations (volume fractions) are plotted against each other, that is, the PEG concentration versus the dextran concentration.^15,16^ The binodal line may be called “open”, and tie lines (lines that can be drawn in the phase diagram that connect the two coexisting points) are running from the top-left to the bottom-right directions (for an example, see Figure 2 below). Water is a natural control parameter. When the water content is low, there are many PEG-dextran contacts and the segregation is relatively strong (little PEG dissolves in the dextran-rich phase and vice versa). With an increasing water content, the number of potential repulsive PEG-dextran contacts decreases, and the two phases become more weakly segregated. At the critical point, the two phases become identical and this point is found somewhere along the binodal in the region where both polymer concentrations are low (and thus the water content is high). (ii) In a second scenario, segregative phase behavior may be triggered by a difference in solvent quality (best seen when the main driving force is absent). The polymer that is most hydrophilic surrounds itself with a bit more water, while the other one chooses the phase with the lower water content. Provided the solvent quality difference is sufficiently large, a “closed” triangular-shaped binodal is found (for an example, see Figure 1 below) that features two critical points, one analogous to the first scenario in the lower left corner of the phase diagram and another one at the upper right side at high polymer–polymer concentrations (low water content). The latter critical point is easily rationalized because in the absence of solvent, the solvent quality cannot have an effect. Hence, upon a decrease in the amount of solvent, the system should again go from a two-phase to the one-phase state. The tie lines are running in the same direction as in the open-binodal scenario (that is, from top-left to bottom-right).

**Figure 1:**
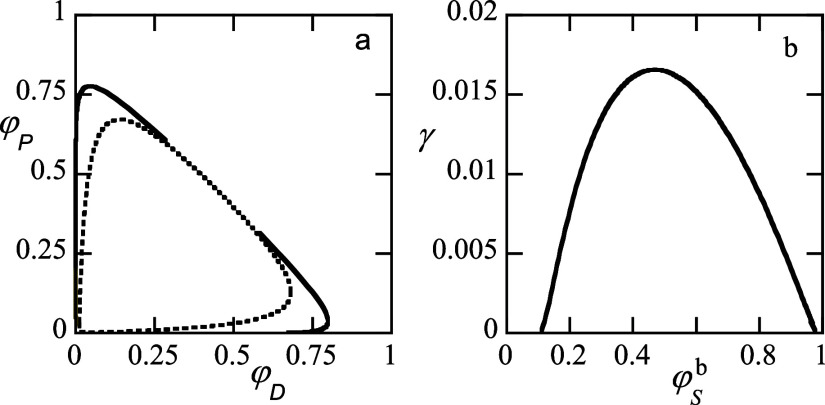
(a) Closed loop binodal φ_*P*_ vs φ_*D*_. Solid line binodal, dotted line is spinodal (obtained analytically from FH-theory). (b) Interfacial tension γ in units of *k*_*B*_ *T*/*b* ^2^ as a function of the volume fraction in the (dextran rich) bulk phase. Default parameters case 2: driving force for segregation is only by solvent quality difference.

**Figure 2:**
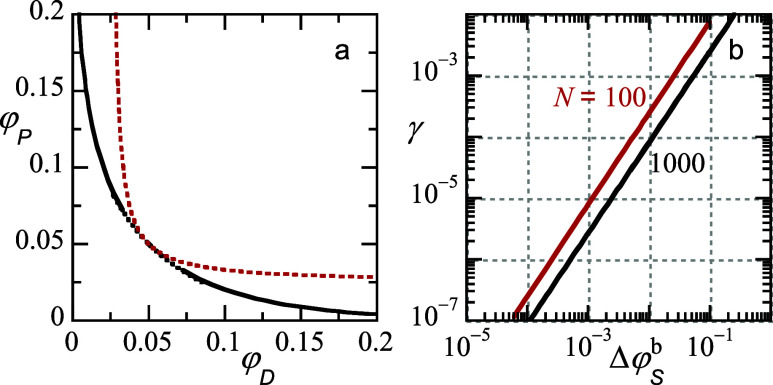
(a) Open binodal φ_*P*_ vs φ_*D*_. Solid line is binodal (dotted black section is the analytical approximation), dotted red line is spinodal (obtained analytically from FH-theory). *N* = *N*_*P*_ = *N*_*D*_ and χ_*PD*_ *N* = 20, binodals and spinodals for *N* = 1000 and *N* = 100 are on top of each other (b) interfacial tension γ in units of *k*_*B*_ *T*/*b* ^2^ as a function of Δφ_*S*_^*b*^ ≡ φ_*S*_^cr^-φ_*S*_^*b*^ in double logarithmic scale. Value for *N* is indicated. Default parameters case 3: driving force for segregation is given by χ_*PD*_ = 0.02 when *N* = 1000 and χ_*PD*_ = 0.2 when *N* = 100 χ_*SP*_ = χ_SD_ = 0.

In experimental situations of PEG-water-dextran systems, it is typically found that both driving forces are operational (cf. Figure 3 below). This is exactly what was found when the experimental phase diagram was fitted by the SF-SCF models.^13^ The experimental binodal is open because both polymers repel each other sufficiently strongly and it was found that a solvent quality disparity enhances the segregative tendency of the system.

**Figure 3:**
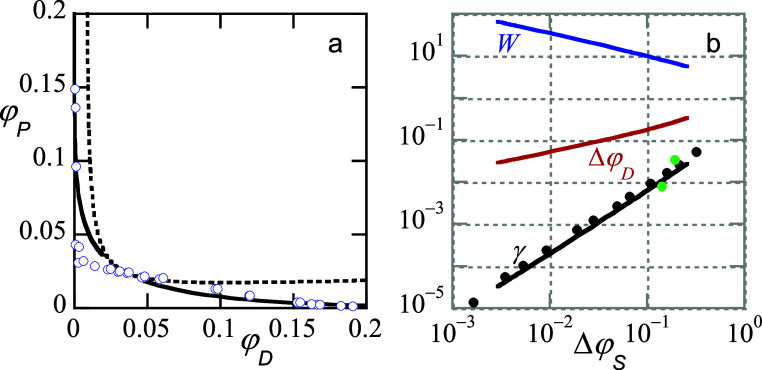
(a) Phase diagram (points) for dextran *M*_w_ 150 000 g/mol, PEG *M*_w_ 20 000 g/mol. SF-SCF fits (binodal solid lines, spinodal dotted line), experimental data (red and blue points are data point measured independently by two individuals). Modeling parameters χ_*DP*_ = 0.2, χ_*DS*_ = 0.48 χ_*PS*_ = 0.44, *N*_*P*_ = 100, *N*_*D*_ = 300. (b) The SCF-prediction for the width *W* of the interface between the dextran-rich and PEG-rich phases, the corresponding density difference Δφ, the interfacial tension γ (in units of *k*_*B*_ *T*/*b* ^2^) (*b* is segment length) as a function of Δφ_*S*_, with critical value φ_*S*_^cr^ ≈ 0.95 in double logarithmic units. The scaling results are in line with van der Waals theory.^31,35^ On the interfacial tension curve the points represent measurements with the spinning drop method and presented in dimensionless units. The green dots are for the same system as presented in panel (a). The black dots are for a slightly different system from the literature,^36^ i.e. dextran *M*_w_ between 300 and 400 kg/mol and PEG *M*_w_ 7 kg/mol. For the fits we needed conversion form concentration to volume fractions. For simplicity, we used that the density of PEG and the density of dextran were similar to that of water.

When solid surfaces are in the presence of a PEG-dextran solution with a solubility gap, we clearly have three phases: (i) the solid, (ii) the PEG, and (iii) the dextran-rich one. Then there can be up to three interfaces: (i) one between the solid and dextran-rich phases, (ii) one between the solid- and PEG-rich phases, and (iii) one between the dextran-rich and PEG-rich phases. If all three interfaces are present, one must have a three-phase contact line and a finite contact angle that characterizes how these phases come together in a partial-wetting situation. Alternatively, there are only two interfaces:^17^ There is an interface between the dextran-rich and PEG-rich phases, but the solid/PEG-rich or the solid/dextran-rich interface is missing. The solid surface is said to be (completely) wet by the dextran-rich phase or the PEG-rich phase, respectively (and the solid is dry with respect to the other phase). With a suitable control parameter (water is a natural choice), one can force a wetting transition in which the system goes from a partially wet to the completely wet (or dry) state.

There are several accepted and equivalent routes to study wetting scenarios.^17,18^ Here we focus on analyzing adsorption isotherms because these are easily understood and informative regarding the order and location of wetting transitions. Our focus is in general on the adsorption of the wetting component (in our case PEG), as a function of the logarithm of the (bulk) concentration of PEG (at some fixed volume fraction of water and (thus) a variable concentration of dextran). The features of such isotherms in the vicinity of the bulk saturation (binodal value) of PEG is essential. In all cases the isotherm diverges at the bulk binodal, but how exactly this limit is reached characterizes the wetting scenario. If with increasing bulk concentrations the isotherm shows a monotonic increase of the adsorbed amount, we know that the surface is wet by this component. Alternatively, the isotherms may show a “loop” in the sense that the bulk concentration hits the binodal value at a very low adsorbed amount and a part of the isotherm exists in the supersaturated concentration domain before it eventually returns to the binodal value. The system may be characterized as partially wet or completely wet, depending on the grand potential values for the first crossing with respect to the limiting grand potential at the top of the isotherm. If this value is lower at the first crossing, then the system is in a partially wet condition and the contact angle is finite. Inversely, when the value at the first crossing is the higher value, the system is characterized as “wet” and the contact angle is zero. Exactly at the wetting transition there exists an equal-area argument to localize the transition and this transition point is at the binodal.

Upon a suitable control parameter (e.g., the amount of water in the system), one can trigger wetting transitions, e.g. from partial to complete wetting. These will be first-order (jump-like) or second-order (smooth, no jumps).^17,19^ In the latter scenario, the adsorbed amount for the first crossing of the binodal continuously increases upon the approach of the wetting transition and diverges at the wetting transition. In the first-order case, the excess amount at the surface (adsorbed amount) at the first crossing remains finite below the wetting transition and jumps to infinity at the wetting transition. This information is obtained from the shape of a family of adsorption isotherms. When the adsorption strengths are fixed, we can use the water content as the main control parameter to generate relevant isotherms. Alternatively, at fixed (bulk) water concentration, we use a variation of the appropriate interaction parameters for the surface to do the same. In the first-order wetting scenario, there is a loop in the isotherm. When this loop occurs before the binodal is reached, the jump in adsorbed amount (located by the equal area argument) is known as the prewetting transition. In a second-order wetting transition, the prewetting step is missing.

Evidently, adsorption or depletion of the PEG/dextran components are mechanistically relevant for wetting, while the solvent plays only a secondary role.^20^ Polymer adsorption from low molecular weight solvents is a classical field of research. It is relevant to briefly mention a few aspects of polymers at interfaces.

For polymers near interfaces, the concept of critical adsorption energy is important. This notion reflects the fact that adsorbed polymers experience a loss in conformational entropy.^20^ To compensate for this, there must be a finite adsorption energy before the chains have a tendency to adsorb. The loss of entropy per segment is (in a lattice model with coordination number *Z* = 6) estimated as Δ*S* = *k*_*B*_ ln 6/5, resulting in a critical adsorption energy of approximately −0.15 *k*_*B*_ *T* (in terms of the interaction parameters^21^ used below, this gives χ_*WX*_^cr^ ≈ −1 where *W* refers to the surface and *X* a polymer segment type).

The notion of the cooperative nature of polymer adsorption has received a great deal of attention in the literature.^20^ This explains, for example, the preferential adsorption of long polymers over shorter ones when the segmental affinities and concentrations are similar. We know that a low molecular weight molecule (displacer) can displace long adsorbed polymers from the surface when the adsorption energy of the displacer is higher than that of the polymer segments and when the displacer concentration increases.^22–25^ A systematic survey of how one polymer type displaces the other is largely missing. More specifically, the type of displacement transition (first/second order or a smooth one) is not yet discussed in the literature. It was found that the process of polymer displacement could be very slow, i.e., it can take up to many hours.^20^ Such slow kinetics is consistent with metastable states that can exist when the adsorption displacement transition occurs as a phase transition, but it is not conclusive evidence for it. More specifically, in segregative conditions, we do not know of any analysis of how polymer displacement occurs. We will show that in the absence of segregative driving forces, the displacement process is smooth. However, subject to segregative driving forces and when the dextran chains have a finite affinity for the surface, while the PEG chains are bound even stronger, the displacement by PEG chains may become a first-order transition.^19^ We will elaborate on how the displacement transitions and the (first-order) prewetting transition influence each other.

In a seminal paper, Nakanishi and Fisher^19^ report a detailed survey of surface phase transitions extracted from a phenomenological Landau free energy functional. This approach is well-known to cover wetting transitions: a surface phase transition occurs while the bulk is at its binodal (see above). However, they also envisioned a pure surface phase transition when the surface “enhancement” is sufficiently strong while the surface “field” and, importantly, the bulk “field” both being small or even zero. In such scenario, the bulk remains in a one-phase state even when at the surface a phase change is triggered. In the following, we will show that in some cases the polymer displacement transition can manifest itself as a pure surface phase transition, while in other cases it shows up as a prewetting transition.

In our previous paper, we performed a self-consistent field analysis of segregative aqueous dextran-polyethylene glycol solutions focusing on bulk phase behavior.^13^ In that study, we established modeling parameters for the experimental system that is investigated in our own laboratory.^6^ One of the aims of the current analysis is to scrutinize the wetting characteristics in this experimental system. Predictions rely invariably on the parameters found from our bulk study, and only the parameters that characterize the surface interactions will be added to this. To get generic insight and to see the main trends for wetting phenomena in ATPS, it is useful to first focus on simpler systems and avoid complications. More specifically, we will choose an idealized parameter set, albeit that these typically will remain close to the experimentally relevant set.

The remainder of this paper is as follows. We will start with a methodology section, wherein the SF-SCF approach is discussed focusing on the main approximations and characteristics, explaining what input quantities are needed and what comes out of the modeling in terms of results and observables (with a main emphasis on adsorption and wetting related quantities). For a more detailed overview of the approach, we refer to the literature^20,26–28^ or the Supporting Information of the previous paper in this series.^13^ In the Parameter Section, we pay attention to the interaction parameters and give the rationale for the choices that are implemented. Here we will also highlight the parameters that were found for our experimental system. In the “Results and Discussion” we will focus on the displacement transitions that may manifest as a prewetting transition or as a pure surface phase transition. We do this in close connection with a wetting study. In the final parts of the Results and Discussion section, we will focus on the experimental system and highlight why in the far majority of situations we expect that the PEG-rich phase completely wets the particles. Conclusions will be stated at the end, as usual.

## 2 Methodology

When a system features three phases, one should be aware of wetting phenomena.^17^ The generic nomenclature is that there is a liquid (L) sitting on top of a substrate (S) in a surrounding vapor (V). There are three interfacial free energies γ_LV_, γ_SV_ and γ_SL_ in the Young–Laplace equation^18,19,29^

1 $$\cos\theta =\frac{\gamma_{SV}-\gamma_{SL}}{\gamma_{LV}}$$

2 $$S\equiv \cos\theta -1=\frac{\gamma_{SV}-\left(\gamma_{SL}+\gamma_{LV}\right)}{\gamma_{LV}}$$

3 $$S=\frac{\gamma_{thin}-\gamma_{thick}}{\gamma_{LV}}$$

where θ is the contact angle (through the liquid) that characterizes how the liquid drop sits at the solid substrate in the presence of a surrounding vapor phase. When 0° < θ < 180°, we talk about partial wetting. When θ = 0° the surface is wet, whereas in the other limit θ = 180° the surface is dry. Focusing on the transition from partially wet to completely wet, this happens when the spreading parameter *S* changes sign. This occurs when γ_thin_ = γ_thick_. The keywords “thin”and “thick” refer to the quantities that can be extracted from so-called adsorption isotherms in SF-SCF calculations, specifically referring to the thickness of the layer of the wetting phase when the adsorption isotherm crosses the binodal for the first time (this film is “thin”) and when the adsorption isotherm approaches the binodal where the adsorption diverges (this film is “thick”). In the literature, the spreading parameter is often defined by *S*≡γ_thin_–γ_thick_ especially if the interest is only in the sign switch of *S*.^18^

Focusing on the classical Young–Laplace eq 1, we can already learn that a very low γ_LV_ may give cos θ → ± 1 even when the difference |γ_SV_–γ_SL_| is small. This is particularly relevant in ATPS which are known for the very low value of the interfacial tension between the PEG-rich and dextran-rich phases (here given by γ_LV_). Indeed, this notion is known as the Cahn conjecture^30^ and is also known as critical point wetting (CPW); Cahn argued that upon the change of a suitable control parameter (for example the temperature or in our case, the amount of solvent), a system will first witness a wetting transition from partially to completely wet (or dry) before it undergoes a bulk phase transition from a two-phase to a one-phase system. The reason for this is that upon the approach toward the bulk critical point, the difference |γ_SV_–γ_SL_|decreases more slowly than the value of γ_LV_. In the following, we will refer to the Cahn conjecture multiple times to explain the wetting trends in our ATPS. For example, due to the low value of γ_LV_, we expect ATPS systems to preferentially solubilize particles in either the PEG-rich or dextran-rich phase, while cases where particles are at the interface between the PEG-rich and dextran-rich phases should be rare. It remains of interest to determine the parameters that correspond to these rare situations.

When polymers adsorb spontaneously on surfaces, the interfacial free energies that correspond to these surfaces will decrease. A higher affinity implies more adsorption and a greater reduction of these interfacial (free) energies. Hence, the link between the adsorption and wetting properties of the system is quite obvious. A suitable theory to investigate wetting in ATPS should therefore both be able to give insight in the structure of the adsorption layer and relate this to the corresponding interfacial thermodynamics. Importantly, the theory should give access to interfacial free energies, especially when the chemical potentials of all components of the system are at the so-called binodal values.^31^ A suitable method should obviously be able to cope with the (bulk) phase behavior and be able to evaluate the relevant interfacial tension.

Predicting free energies, including interfacial free energies by computer simulations is challenging,^32^ which makes these approaches less attractive for the present study. The SF-SCF approach,^13,20^ described below, is a method that has been used before to predict the wetting effects of molecularly complex systems. It gives numerically accurate predictions for the adsorption characteristics and (interfacial) free energies. It accounts for the bulk phase behavior, etc., albeit that all results are of the mean-field type. It is argued that for polymeric systems the mean field predictions are reasonable, arguably less when systems are very close to its critical point(s), when fluctuations are important. In these limits, the theory has only a qualitative accuracy. The SF-SCF approach is lattice based and has approximations similar to the well-known Flory–Huggins (FH) theory.^21^ The FH-approach can be used to give the bulk phase behavior, and that is why we will start with the FH-theory before SF-SCF is outlined.

### 2.1 Modeling ATPS: Bulk

Long polymers have many universal properties and this is why coarse-grained models in which chemical details are lumped in statistical segments may be used to investigate them. The Flory–Huggins approach is a key example of such a model and is a useful starting point to study the bulk phase behavior. The Flory–Huggins theory^21^ makes use of lattice approximations and thus ignores the chemical details on the level of segments: both polymeric segments and solvent molecules are of the size of a lattice site and are assumed to mix randomly throughout the system. Focusing on a system with two polymeric components, where *D* represents dextran and *P* represents PEG in a monomeric solvent S (e.g., water), the Flory–Huggins (dimensionless) free energy density reads

4 $$f=\frac{\varphi_{D}}{N_{D}}\ln\varphi_{D}+\frac{\varphi_{P}}{N_{P}}\ln\varphi_{P}+\varphi_{S}\;\ln\varphi_{S}+\chi_{DP}\varphi_{D}\varphi_{P}+\chi_{DS}\varphi_{D}\varphi_{S}+\chi_{PS}\varphi_{P}\varphi_{S}$$

here, *N*_*D*_, and *N*_*P*_, are the degree of polymerization of dextran and PEG, respectively (here modeled by the number of lattice sites occupied by the macromolecule). Interactions are accounted for by the Bragg-Williams approximation and parametrized by the Flory–Huggins interaction parameter

5 $$\chi_{AB}=\frac{Z}{2K_{B}T}\left(2U_{AB}-U_{AA}-U_{BB}\right)$$

with *k*_*B*_ *T* is the thermal energy, *Z* the lattice coordination number (*Z* = 6 for cubic lattice, *Z* = 4 hexagonal lattice), and *U*_*AB*_ is the depth of the interaction potential for the square well potential accounting for nearest neighbor *A*-*B* contacts. Similarly, *U*_*AA*_ accounts for *A*–*A* and *U*_*BB*_ for *B*–*B* contacts. In eq 4 φ is a volume fraction. The free energy density eq 4 appears to be a function of three volume fractions φ_*D*_, φ_*P*_ and φ_*S*_. However, in an incompressible system we can reduce this by one: i.e., φ_*S*_ = 1–φ_*P*_–φ_*D*_. There are analytical solutions for the spinodals, whereas the binodals can only be found numerically. However, the FH-method is not a suitable starting point to investigate the properties of interfaces between these bulk phases, and therefore the SF-SCF method is used instead.

### 2.2 Modeling ATPS: Interface

Scheutjens and Fleer (SF) proposed a free energy functional^26,27^ that feature volume fraction profiles φ_*X*_(*z*) and complementary segment potential profiles *u*_*X*_(*z*) for all segment types X = *D*, *P*, *S*, targeted to study polymers at interfaces. Here the *z*-coordinate is chosen to run normal to the interfaces laying in the *x*–*y* plane. As *x* and *y* do not occur in the free energy functional this implies that the densities are averaged over these coordinates. Hence, the three-dimensional problem is reduced to a one-gradient mean-field model. These authors advocated to use a freely jointed chain model to account for the (conformational) entropy of polymers at interfaces. They also proposed to account for interactions similarly as in Flory–Huggins theory, with the notable difference that so-called site-fractions are introduced to make sure that in “gradients” the contact-counting remains accurate. (This is equivalent to the square gradient term in Cahn Hilliard theory^33^).

A saddle-point analysis of the free-energy functional leads to the self-consistent field rules. One rule specifies how the segment potentials follow from the volume fractions. Another rule specifies how the volume fractions must be computed from the segment potentials. The so-called propagator formalism implements this rule for the freely jointed chain (FJC) model. In this model two consecutive segments along the chain occupy neighboring segments on the lattice, but longer-ranged correlations are ignored. This implies that chain back folding is not forbidden (intramolecular excluded-volume problem), nor are segments of different chains forbidden to occupy the same site (intermolecular excluded-volume problem). An incompressibility condition sets a constraint on the volume fractions in each coordinate *z*: φ_*D*_(*z*) + φ_*P*_(*z*) + φ_*S*_(*z*) = 1, which partially corrects for possible inter- and intramolecular excluded-volume errors. In summary the three rules are

6 rule1:potentialsfollowfromdensities:u[φ]

7 rule2:densitiesfollowfrompotentials:φ[u]

8 $$rule3:incompressible:\sum_{X}\varphi_{X}\left(z\right)=1\forall z$$

The volume fraction profiles and segment potential profiles that obey to all rules simultaneously, characterize the self-consistent field (SCF) solution. These are found numerically using an iterative procedure. Such SCF solutions are reached routinely with an accuracies of 9 significant digits. The resulting profiles may be inserted in the free energy functional to generate relevant thermodynamics corresponding to equilibrium. Again, the predicted bulk phases are exactly the same as those found by FH theory. So, the numerical values for the chemical potentials of all molecular species of the system are available from the FH-theory and therefore, the grand potential Ω defined by Ω = *F*–*∑*_*i*_μ_*i*_ *n*_*i*_, where the *i*-index refers to the molecule type (dextran, PEG or solvent), *n* is the number of molecules (per lattice site, effectively per unit area) μ the chemical potential and *F* the free energy (per unit area of the system) is routinely evaluated. It turns out that Ω = ∑_z_ω­(*z*) where the grand potential density ω­(*z*) is given in closed form^28^

9 $$\omega \left(z\right)=-\sum_{i}\frac{\varphi_{i}\left(z\right)-\varphi_{i}^{b}}{N_{i}}-\alpha \left(z\right)-\frac{1}{2}\sum_{A}\sum_{B}\chi_{AB}\left[\varphi_{A}\left(z\right)\left\langle \varphi_{B}\left(z\right)\right\rangle -\varphi_{A}^{b}\varphi_{B}^{b}\right]$$

where the indices *A* and *B* run over the three segment types *D*, *P*, *S*, α­(*z*) is the Lagrange field contribution to the segment potential at coordinate *z*, its value is tuned such that the system obeys to the incompressibility constraint at this coordinate. The angular brackets implement the site-fraction mentioned above: $\left\langle \varphi \left(z\right)\right\rangle =\frac{1}{Z}\left(\varphi \left(z-1\right)+\left(Z-2\right)\varphi \left(z\right)+\varphi \left(z+1\right)\right)\approx \varphi \left(z\right)+\frac{1}{Z}\frac{\partial^{2}\varphi \left(z\right)}{\partial z^{2}}$ .

For the liquid–liquid (L–V) interface, Ω is identified as the liquid–liquid interfacial tension γ_LV_. In an ATPS the interfacial tension between the PEG and dextran-rich phases is specified by γ_LV_≡γ_DP_.

The SF-SCF equations must be solved using appropriate boundary conditions. Reflecting boundaries are applied at the lower (*z* = 0) and the upper boundary of the system (*z* = *M*) when bulk phase behavior is probed. In all cases, the value of *M* (system size) is chosen such that it exceeds the width of the interface several times. The interface is then preferably positioned halfway between upper and lower limits of the lattice, and the boundaries do not affect any of the interfacial properties. Alternatively, our interest may be in features near a solid interface. In this case the boundary condition at the upper bound (reflecting) remains unaltered, while the boundary condition at *z* = 0 is changed to adsorbing: a solid component *W* is introduced that has a “frozen” (that is unchangeable) step-like, density profile

10 $$\varphi_{W}\left(z\right)=\{\begin{array}{ll} 1 & \text{if}z<1\\ 0 & \text{if}z>0 \end{array}$$

The number of polymer surface contacts is evaluated using the site-fraction method and the contact energies are parametrized by χ_DW_, χ_PW_ and χ_SW_. It is customary to set χ_SW_ = 0 and then the adsorption energy parameters of DW and PW are considered to have values that reflect the adsorption strength as compared to that of the solvent.

In adsorption isotherms we collect the excess amount of a given component *i*, θ_i_^σ^ as a function of the bulk concentration of this component φ_*i*_^*b*^. In this case, the bulk is defined as the (majority) phase that exists far from the surface. More specifically, we are typically interested in the component (*i*) that has the highest affinity for the surface *W* so that the excess given by

11 $$\theta_{i}^{\sigma }=\sum_{z=1}^{M}\left(\varphi_{i}\left(z\right)-\varphi_{i}^{b}\right)$$

is positive. We will see examples of adsorption isotherms in the case of ATPS for which *i* = PEG (*P*). At each point along the adsorption isotherm (θ_P_^σ^(φ_*P*_^*b*^) it is possible to compute the grand potential Ω. Thus, we can record Ω­(φ_*P*_^*b*^). In wetting studies, the values of Ω when the bulk concentration reaches the bulk binodal value φ_*P*_^*b*^ = φ_*P*_^#^ are used to predict the contact angle.

In the ATPS case, as explained above, the relevant isotherms have in common that the value of θ_P_^σ^ eventually diverges (there exists an arbitrarily thick film of the *P*-rich phase at the surface) and φ_*P*_^*b*^ reaches the bulk binodal value φ_*P*_^#^. In this limit Ω­(φ_*P*_^#^) may be identified by γ_thick_. In the case of partial wetting, the isotherms will cross the binodal bulk concentration, φ_*P*_^#^, already at relatively low values of θ_P_^σ^, that is, when the adsorption layer is still relatively thin. At the first crossing of the binodal value, we can identify γ_thin_ ≡ Ω­(φ_*P*_^#^). As mentioned above (cf., eq 3) γ_thin_ and γ_thick_ are used to evaluate the contact angle. The value of γ_DP_ is available from the analysis of the interface between the dextran-rich and PEG-rich phases (a separate calculation).

### 2.3 Parameters and Bulk Phase Behavior

In the results section below, we basically cover four cases:^13^ (i) The ideal system, (ii) the closed-loop binodal system, (iii) the open binodal system, (iv) our experimental case study. The default parameters in these four cases are listed and elaborated briefly in the current section and corresponding bulk phase behavior is reported. It is impossible to cover all possible parameter settings. We trust that by presenting these four cases a comprehensive picture emerges.

#### 2.3.1 Ideal Case

     When the solvent quality is the same for both polymers and when the two polymers mix ideally, we have a ternary system that has no tendency to segregate. Although the ideal case must be the exception, it remains important to study. As there is no tendency to segregate, we have no wetting issues and our primary interest is to unravel the adsorption preference of one polymer over the other. For the default reference system, we take both polymers with the same degree of polymerization *N*_*P*_ = *N*_*D*_ = 1000, both polymers have the same (strong) interactions with the surface χ_WD_ = χ_WP_ = – 4, while other parameters are taken to have the athermal value χ_PD_ = χ_SP_ = χ_SD_ = 0. Then, when both polymers also have the same bulk concentration, we must expect PEG- and dextran chains to populate the surface equally. Quite arbitrarily, we fixed the bulk volume fraction of dextran to 10^–3^ (that is, near the overlap concentration).

#### 2.3.2 Closed Loop Binodal; Minor Driving Force

     Deviating from the ideal case, we will first consider the case where the polymers differ with respect to their affinity for the solvent only. According to our bulk study^13^ this corresponds to the “minor driving force” case, which features a closed-loop binodal.

A typical example will be considered: Both polymers are taken to be equally long *N*_*P*_ = *N*_*D*_ = 1000. Importantly, there is no repulsion between the two segments χ_*DP*_ = 0 (that is, there is no major driving force for segregation). Inspired by experimental trends, we take theta-solvent conditions for dextran χ_*DS*_ = 0.5 and marginal solvent conditions for PEG χ_*PS*_ = 0.3. This difference in solvent quality is sufficient to have segregative phase behavior. A larger solvent quality disparity will increase the solubility gap and a smaller difference will decrease it.

The corresponding closed-loop phase diagram in terms of the volume fraction of PEG and the volume fraction of dextran is shown in Figure 1a by the solid lines. Because of the rather large difference in solvent quality and the use of relatively long chains, the binodal has a semitriangular shape. The tie lines (not shown) run with a slope close to −1. There are two critical points, one in the lower left corner and another one at a volume fraction φ_*P*_ ≈ φ_*D*_ ≈ 0.495. The spinodals (dotted line) were found analytically from the Flory–Huggins theory eq 4 by finding the roots of the free energy Hessian

12 $$f_{DD}f_{PP}-f_{DP}^{2}=0$$

where *f*_*XY*_≡∂^2^ *f*/∂φ_*X*_∂φ_*Y*_.

In the range 0.1<φ_*S*_^*b*^ < 0.94 the system is in the segregated state. The corresponding interfacial tension as a function of the solvent volume fraction in the dextran-rich phase is given in Figure 1b. As expected, the tension vanishes at the two critical points and passes through a maximum at some intermediate solvent volume fraction. The conversion of dimensionless interfacial tensions to N/m is given by *k*_*B*_ *T*/*b* ^2^ ≈ 16 mN/m, where the segment size *b* = 5 × 10^–10^m has been used. This means that a dimensionless interfacial tension of unity translates to a tension of 16 mN/m. Hence, the interfacial tension in these systems is, characteristically for ATPS, extraordinary low, that is, significantly less than 1 mN/m.

We expect that both dextran- and PEG chains have a sufficient affinity for the silica surface to (strongly) adsorb onto it.^23^ However, as indicated by the experimental observation that the silica particles partition in the PEG-rich phase, we conclude that PEG has a higher affinity for the (silica) surface. As long as there is a large adsorption preference of PEG-chains over dextran ones, it is found that the surface is wet by the PEG-rich phase for all relevant water compositions (consistent with observations). Only when the adsorption preference is small, one should find wetting transitions. For obvious reasons, therefore, we will implement that our reference system has a relatively small (positive) difference between Δχ_*W*_≡χ_*WD*_–χ_*WP*_ = 0.5, which implies that χ_*WP*_ is more negative (higher surface affinity) than χ_*WD*_. The results are complemented by a second parameter setting for which an even smaller difference χ_*WD*_–χ_*WP*_ = 0.1 is chosen. For both parameter sets we may probe the range from strong to weak adsorption. Our interest is in both the polymer displacement transition and the wetting characteristics.

#### 2.3.3 Open Binodal; the Strong Driving Force

     A closed-loop binodal as studied in case 2 must be considered to be a rare case as well. When there are nonideal interactions between the two types of polymer segments, the major driving force manifests.^13^ More specifically, when χ_*PD*_ *N* > 2 (assuming *N*≡*N*_*P*_ = *N*_*D*_ and equal solvent conditions), it becomes impossible to have a closed-loop binodal. In this scenario, we will assume, once again, that both polymers are equally long *N* = 100, and assume the solvent quality to be equally good for both polymers, that is, χ_SP_ = χ_SD_ = 0, implying that the minor driving force is absent. Quite arbitrarily, we set *Nχ*_*PD*_ = 20 so that the major driving force is sufficiently strong and the binodal has the classical shape, i.e., it is not closed. The phase diagram which features a binodal, which separates the 1-phase region from the phase-separated one, is fully symmetric upon the exchange of *P* with *D* (cf. Figure 2a. The critical point for symmetric systems is given by (1 – φ_*S*_)­χ_*PD*_ *N* = 2, which means that for χ_*PD*_ *N* = 20, φ_*P*_^cr^ = *φ*_*D*_^cr^ = 0.05. Near the critical point, there is an analytical approximation for the symmetric binodal

13 $$\varphi_{D}^{bin}=\frac{1-\varphi_{S}^{bin}}{2}\left(1\pm \sqrt{\frac{3}{2}\left(\chi N\left(1-\varphi_{S}^{bin}\right)-2\right)}\right)$$

which is presented by the black dotted line. The dotted red line is the analytical spinodal obtained from Flory–Huggins theory. In passing, we mention that the phase diagram in the symmetric setting depends only on the product *N*χ_*PD*_. For example, there is no difference in the phase diagram for systems with *N* = 1000, χ_*PD*_ = 0.02 and *N* = 100, χ_*PD*_ = 0.2.

The interfacial tension (cf. Figure 2b) follows a power-law dependence $\gamma \propto {\left(\varphi_{S}^{cr}-\varphi_{S}^{b}\right)}^{3/2}$ . The interfacial tension between the dextran-rich and PEG-rich phases is sensitive to the value of *N*. For given χ_*PD*_ *N*, the tension at a specified solvent volume fraction is higher for the system with shorter chains. The 3/2 power-law coefficient is known to be the mean-field value. However, deviations from this value are only expected very close to the critical point.^13,34^

When in this symmetric system the surface affinities are also set to the same values, we have the so-called neutral (wetting) condition. The contact angle will be 90° for all values of the solvent volume fraction. However, a small difference in surface affinity χ_*WP*_ < χ_*WD*_ immediately implies a lower contact angle (here defined with respect to the PEG-rich phase). We will see that partial wetting is only found when there is a small difference in surface affinities for dextran and PEG for the (silica) surface. Upon the change of the solvent volume fraction toward the bulk critical point, the Cahn argument suggests that a wetting transition will take place. By far, most parameter settings result in the case that the system is wet by either PEG-rich phase or the dextran-rich phase. Inspection of Young’s law (cf. eq 1) shows that a low interfacial tension (cf. Figure 2b) is the cause of this. Hence, the window of partial wetting for the system with *N* = 1000 is smaller than that for *N* = 100.

#### 2.3.4 Experimental Case Study

     In our laboratory, we have determined experimentally the phase diagram for a PEG-dextran-water system (in the caption of Figure 3 you will find detailed information). We have used the SF-SCF model to fit this phase diagram using a simplified model.^13^ Although the experimental system is polydisperse, for the SF-SCF computations a binary (monodisperse) model with *N*_*P*_ = 100 and *N*_*D*_ = 300 was implemented. The effects of polydispersity on the phase diagram (cf. Figure 3a) are most clearly noticed near the critical point, and this explains the mismatch between theory and experiments in this region of the interface to a large extent. As can be seen in Figure 3a, the phase diagram is sufficiently asymmetric. This is caused both by the difference in lengths of PEG and dextran chains and by the solvent quality difference. The dextran is expected to be around theta conditions χ_*DS*_ = 0.48, while the solvent quality of PEG is slightly better, χ_*PS*_ = 0.44. The interaction between PEG and dextran was fitted to χ_*PD*_ = 0.2. We have an estimate for the critical volume fraction of solvent in this system, that is φ_*S*_^cr^ ≈ 0.95. Experimentally, the critical point is less certain mainly because polydispersity effects become apparent near the critical point. The highest total polymer concentration that can practically be reached is approximately 35 weight %, and we interpreted this as an overall volume fraction of polymer of φ = 0.35.

Experimentally, we have studied both silica and polystyrene particles dispersed in ATPS. Even at very low water compositions, we found that the particles dispersed in the PEG-rich phase, indicating complete wetting up to low water content. After vigorously mixing the PEG-dextran-particle system and then left to relax, evolves smoothly to macroscopic phase separation, showing that Pickering emulsions did not form. This also indicated to us that the experimental system is in the completely wet state. The SCF analysis of the experimental system focuses on the wetting and drying transitions in the experimental system. These results give straightforward access to the parameters for which partial wetting-, complete wetting (of PEG-rich phase) and complete drying (i.e., complete wetting of the dextran-rich phase) are expected.

## 3 Results and Discussion

### 3.1 Ideal Case

We will start to illustrate that the displacement of adsorbed dextran chains by PEG is a smooth transition as long as the solution is ideal in the sense that the dextran and PEG chains have athermal interactions and equal solvent qualities. Both mentioned driving forces for segregative phase separation are absent and hence the solution will remain in a one-phase state over the whole concentration range.

In Figure 4a three examples are presented for displacement isotherms in the absence of a segregative tendency. It is shown that upon an increase of the PEG concentration in the system, the adsorbed amount of PEG increases (solid line), while that of dextran chains decreases with (roughly) the same amount (dotted line).

**Figure 4:**
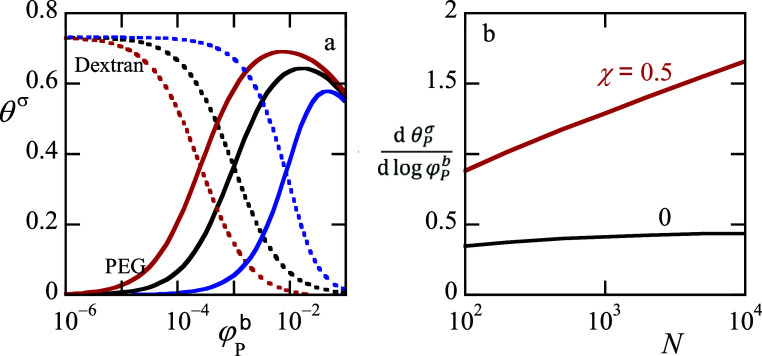
(a) The excess adsorbed amount of dextran (dotted lines) and the corresponding excess amount of PEG (solid lines) as a function of the bulk volume fraction of PEG φ_*P*_^*b*^ while φ_*D*_^*b*^ = 10^–3^. Black curves: default parameters are given in parameters section case 1; blue curves: default except *N*_*P*_ = 500, red curves: default except χ_*WP*_ = – 4.05 (b) the slope of the displacement isotherm θ_P_^σ^(log­(φ_*P*_^*b*^)) at φ_*P*_^*b*^ = φ_*D*_^*b*^ as a function of the chain length *N*≡*N*_*P*_ = *N*_*D*_, while the volume fraction of dextran is fixed to φ_*D*_^*b*^ = 1/*N*_*D*_ (at overlap concentration). Other parameters case 1 defaults.

Focusing first on the black lines in panel Figure 4a we have the (default case 1) symmetric case, (both polymers are equally long, *N*_*P*_ = *N*_*D*_ = 1000, and both polymers have the same affinity with the surface (χ_*WP*_ = χ_*WD*_ = – 4, which for the hexagonal lattice implies that each surface contact gives 1 *k*_*B*_ *T* adsorption energy and both polymers are in the strong adsorption regime). In this regime, the adsorbed amount is in the plateau of the adsorption isotherm (θ^σ^ ≈ 0.73). When the excess amount of PEG increases (because of the increase in the PEG bulk volume fraction, that of dextran goes down because both chains compete for the surface. As explained by symmetry, exactly at φ_*P*_^*b*^ = 10^–3^ the excess adsorbed amount of PEG equals that of dextran. Upon further increase of the bulk concentration (well above the overlap concentration), the excess of *P* passes a maximum. The reason for this is rather trivial. When the polymer bulk volume fraction approaches unity, the excess necessarily drops to zero. Importantly, we see that the displacement of dextran chains by PEG ones is a smooth transition that takes several decades in concentration to complete.

Before we elaborate on the other two results, we first turn our attention to Figure 4b. In this plot, we present the slope of the displacement isotherm as a function of the length of the two polymers (*N*_*P*_ = *N*_*D*_), taken at the location where both polymers have the same bulk volume fraction (that is, halfway through the displacement process). Results for both good solvent and for theta-solvent conditions are shown. The increase of this slope as a function of the chain length means that the displacement transition becomes more cooperative. Indeed in θ-solvent the slopes are a factor of 3 higher than in good solvent conditions. Importantly, the slope remains finite even for very large *N*, and we conclude that the transition is smooth (there is no coexistence of phases).

Returning to Figure 4a, it is seen that when the PEG chains are shorter than the dextran ones (blue curves) the transition occurs at relatively high PEG bulk volume fractions. As shown by the red curves, which give the result for the case that the PEG chains are slightly more strongly adsorbing, the adsorption displacement occurs at relatively low bulk volume fraction of PEG. In both cases, the displacement transition remains smooth.

### 3.2 Minor Driving Force­(Closed Loop Binodal)

The ideal parameter setting is quite academic and therefore we now turn to segregative conditions that lead to ATPS (cf., Figure 1a) and discuss how the displacement of adsorbed dextran by PEG occurs in such systems. This phenomenon is complicated by the fact that we need to be on the outlook for wetting features as well. More specifically, our focus is to show how a prewetting transition may interfere with the adsorption displacement process.

As mentioned above, we now choose conditions such that dextran adsorbs less strongly to the surface than PEG while the difference in surface affinity Δχ_*W*_≡χ_*WD*_–χ_*WP*_ = 0.5. Importantly, we take χ_*PD*_ = 0, implying that the major driving force for segregation is absent, while the only segregative driving force is a solvent quality disparity (minor driving force). Both chains are taken to be equally long (*N* = 1000).

The adsorption isotherms for PEG and dextran are shown in Figure 5a,d for a weak and a strong adsorption case, respectively. They are computed with the constraint that the volume fraction of water in the dextran-rich phase is fixed to a specified value. Hence, both the bulk volume fraction of dextran and that of PEG can vary (when one increases, the other necessarily decreases).

**Figure 5:**
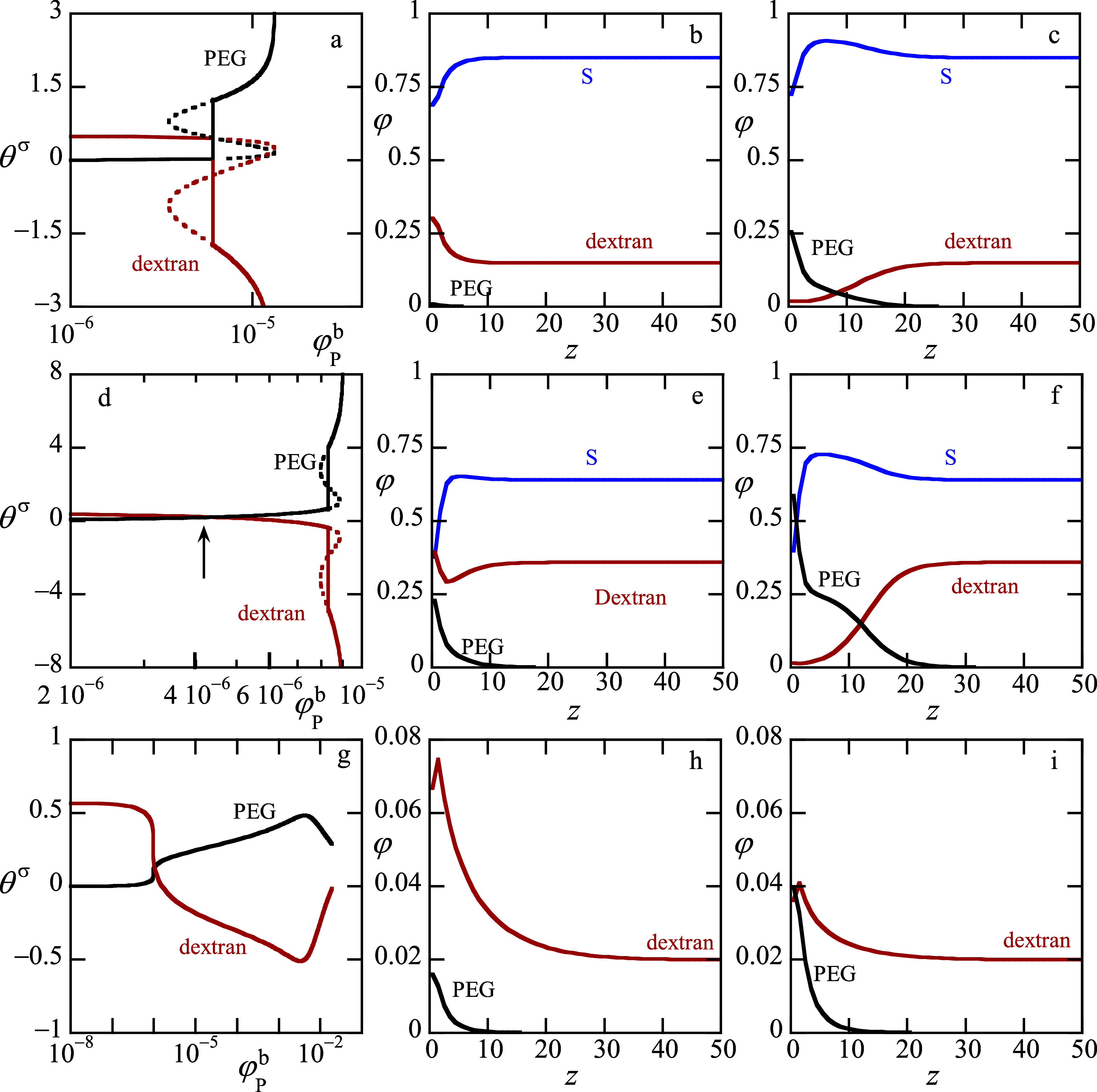
(a,d,g) Adsorption isotherms, the excess amount as a function of PEG bulk volume fraction, of PEG (black curve) and dextran (red curve) (θ_P_^σ^(φ_*P*_^*b*^),θ_D_^σ^(φ_*P*_^*b*^)),b,c,e,f,h,i) Polymer and solvent volume fraction profiles φ­(*z*), where *z* is the distance to the surface in lattice units wherein solvent (blue), dextran (red) and PEG (black) lines. Profiles (b,c, and e,f and h,i) coexist. (a,b,c) weak adsorption: χ_*WD*_ = – 1.5, χ_*WP*_ = – 2 and the solvent volume fraction in the dextran rich phase fixed to φ_*S*_^*b*^ = 0.85 (d,e,f) strong adsorption χ_*WD*_ = – 3, χ_*WP*_ = – 3.5, φ_*S*_^*b*^ = 0.64 (b,e) profiles for the thin film, (c,f) profiles for mesoscopically thin film. g,h,i supercritical case with jump-like displacement transition. χ_*WD*_ = – 0.9, χ_*WP*_ = – 1.4, fixed volume fraction of solvent φ_*S*_^*b*^ = 0.98.Other parameters are given in parameter section: case 2. The arrow in panel *d* points to the (continuous) displacement transition, where dextran chains are replaced by PEG ones near the surface.

For both adsorption strengths, the isotherms indeed have the characteristic features of a system that is in the completely wet state: the adsorbed amount of the wetting component PEG (black lines) diverges upon the approach of the binodal value (in the weak adsorption case (panel a), the binodal is at φ_*P*_^*b*^ ≈ 1.33 × 10^–5^, whereas in the strong adsorption case (panel d), the binodal is at φ_*P*_^*b*^ ≈ 8.99 × 10^–6^). In both examples, there is a first-order wetting transition. This can be concluded from the fact that there exists a prewetting step in both isotherms. In the case of weak adsorption (panel a), the prewetting step is at φ_*P*_^*b*^ ≈ 5.9 × 10^–6^, whereas for strong adsorption (panel d), the prewetting step is at φ_*P*_^*b*^ ≈ 8.3 × 10^–6^ (proximal to the bulk binodal). Whereas the excess of PEG is positive and increases, that for dextran starts off to be positive but turns negative as soon as the PEG-rich phase appears on the surface. Importantly, we notice that in the weak adsorption case (panel a), up to the prewetting step, the dextran chains dominated the adsorbed amount, whereas after that the PEG chains are at the surface. Hence, the displacement transition occurs at the wetting transition step and is thus first-order. In contrast, in the strong adsorption case (panel d), the situation is fundamentally different. Already before the system arrives at the prewetting step in the isotherm, there is a crossover in the dominance of adsorbed dextran chains by PEG ones. This smooth transition is marked by the arrow in panel d. The exact location of this transition may shift when alternative definitions of the surface excess are used, for example as an adsorbed mass per unit area by incorporating the segment molar masses, but the phenomenology will remain the same. Note that in this case there is relatively a small amount of solvent in the system and thus the solvent quality difference presents a weaker force than when the solvent amount is higher. Clearly, when the wetting transition is not far from the upper critical point in the phase diagram (cf. Figure 1), the prewetting step and the crossover in the dominant adsorption species are separate events along the adsorption isotherms. In contrast, when the solvent concentration is high (strong adsorption case), the segregative conditions (solvent quality disparity) are sufficient to have a step-like displacement transition which in this case coincides with the prewetting step.

To better understand what happens at the prewetting step in the isotherm, we present the volume fraction profiles for both the thin and the mesoscopically thick films that coexist (have the same chemical potentials and surface pressures) in panels b, c and c, f of Figure 5 for the weak and strong adsorption cases, respectively. Focusing first on panels b, c (the weak adsorption case), we see that in the thin film the PEG has little or no chains on the surface (panel b), while after the step the PEG chains are the dominant polymer component at the surface. Water likes the PEG chains a bit better than the dextran chains and this explains why the (solvent) *S* component is high where the PEG concentration is relatively high (panel c). The profiles e and f for the strong adsorption cases show the classical result for a prewetting step, where the wetting component goes from a low excess (thin film) to a larger excess (mesoscopically thin). In this process, the film picks up a significant amount of extra solvent, which explains the strong negative excess for dextran. At large values of *z*, i.e. far from the surface, the concentrations of all components in b,c and in e,f approach the same values, as they should because b,c and e,f coexist (have same chemical potentials of all components).

Figure 5g shows the excess amounts of PEG and dextran as a function of the bulk volume fraction of PEG in the case where the amount of solvent exceeds the critical value. Hence, the system is in the supercritical state. Under these conditions, the bulk remains in the one-phase state for all allowed polymer compositions. We recall that there is a significant solvent quality difference χ_SP_ = 0.3 and χ_SD_ = 0.5 and at a high concentration of solvent the displacement of dextran chains by PEG still occurs as a first-order phase transition. Admittedly, the loop in the isotherm is hard to see on the scale of the graph and one is inclined to think that the isotherm corresponds to the surface critical point. However, practically it is almost impossible to hit the critical conditions exactly. In a zoomed-in variant of Figure 5g, in this case one still observes the loop and it is possible to determine the binodal values.

This scenario that there is a jump-like transition even though the bulk remains in a one-phase state, was anticipated by Nakanishi and Fisher^19^ who called this a pure surface phase transition. So, even though the prewetting step stopped to exist (simply because the bulk is supercritical), the displacement transition remains visible as a step in the isotherm. The two density profiles that coexist at the first-order displacement transition are presented in Figure 5h,i. Inspection of these profiles reveals that the excess amount of PEG jumps from a small value to a larger one, whereas the excess of dextran jumps corresponding from a relatively large value to a lower (but still positive) one. Again, as the solvent prefers PEG over dextran, we see that the negative value of the excess dextran occurs at a φ_*S*_^*b*^-value that exceeds the transition value. It is therefore concluded that the polymer displacement transition can be first-order when the segregative driving forces are sufficiently strong yet weak enough so that the bulk remains homogeneous.

As was mentioned above, the signature of a first-order wetting transition is the presence of a prewetting step in the adsorption isotherm. When the step in the isotherm occurs exactly at the bulk binodal, we have identified the wetting transition (WT). For given surface interactions, the amount of solvent in the dextran-rich phase is used as the control parameter to generate such a wetting transition (represented by φ_*S*_^*b*^(WT). Typically, for isotherms with a prewetting step, it is noticed that upon a change of the control parameter to even better wetting conditions, the step in the isotherm occurs progressively away from the bulk binodal. At the same time, the step diminishes in size and eventually vanishes at the so-called surface critical point (SCP) and the value of the control parameter at this surface critical point is denoted by φ_*S*_^*b*^(SCP). In classical wetting theory, such an SCP invariably is found in the vicinity of the wetting transition. A further change of the control parameter to even better wetting conditions leads to adsorption isotherms that no longer have a step and instead monotonically increase and diverge at the bulk saturation value.

In Figure 6 we have collected both values for the control parameter corresponding to the wetting transition (φ_*S*_^*b*^(WT) and that of the surface critical point φ_*S*_^*b*^(SCP) as a function of the affinity of dextran for the surface χ_*WD*_. In (panel a) this is collected for the case that the surface affinity of PEG is higher by 0.5 *k*_*B*_ *T*, Δχ_*W*_ = 0.5, while in (panel b) the difference in surface affinities was set to an even smaller value of Δχ_*W*_ = 0.1. The black lines correspond to the wetting transition. It is seen that the wetting transition remains jump-like (and thus the black line continues) up to the bulk critical point. This is unusual because typically the wetting transition switches from a first-order to a second-order type upon the approach of the critical point. To understand this result, it is useful to discuss the results for the surface critical point (red curves). In between the red and black lines, the isotherms have a step and below the red curves, the isotherms lack the van der Waals loop. It is natural to expect that the SCP occurs at a lower value for the segregative driving force, hence at a lower value for φ_*S*_^*B*^ than that corresponding to the wetting transition (WT). Under subcritical bulk conditions, there is a wetting transition and the SCP is associated with the loss of the prewetting step. However, for supercritical conditions there is no wetting transition and thus there is no prewetting step and hence, we must conclude that the SCP is associated with the polymer displacement transition, which switches from being of a first-order character to a smooth one. Both, the wetting transition and the surface critical point, shift to lower values of φ_*S*_^*b*^ with increasing affinities of the polymers for the surface. This is natural because a high surface affinity will increase the local polymer concentration near the surface and this progressively facilitates the wetting and thus, correspondingly, influences the SCP. Although the difference between the WT and the SCP becomes smaller with increasing affinity for the surfaces, we expect that a difference remains even at stronger affinities than presented in these graphs.

**Figure 6:**
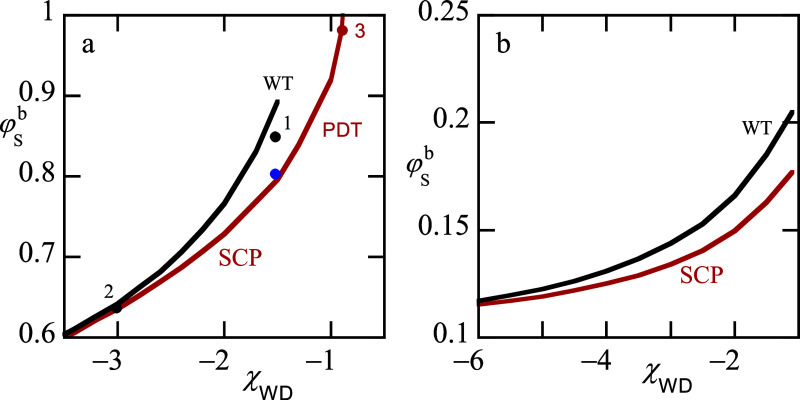
Volume fraction of solvent in the dextran-rich phase (i) black curve, at the Wetting transition (WT) and (ii) red curve at the surface critical point (SCP) versus the adsorption strength for dextran χ_*WD*_. (a) surface affinity for PEG χ_*WP*_ = χ_*WD*_ – 0.5 (b) χ_*WP*_ = χ_*WD*_ – 0.1. In (panel a) the point labeled “1” correspond to the condition of Figure 5a,b,c, point “2” Figure 5d,e,f. The blue point (not numbered) is where the first-order displacement transition goes over to a second-order one (disconnects from the first-order prewetting step). Point “3” linked to Figure 5g,h,i corresponds to the supercritical regime and the SCP which normally is associated with the prewetting step is now associated with the polymer displacement transition (PDT).

Whether the PDT and the prewetting step are separate or combined events still needs to be addressed. In case 2, the only driving force for segregation is a solvent quality disparity. The lower the amount of solvent in the system, the weaker the driving force for segregation. Inversely, the higher the solvent concentration, the stronger the polymers will segregate. In (panel b) for which the adsorption strength difference is small, the wetting transitions were found at very low amounts of water and in all such systems the polymer displacement transition is smooth and detached from the prewetting step. In (panel a) we see 2 scenarios. When the solvent concentration is relatively low, we have the same phenomenology as in (panel b), however, with increasing amounts of solvent, the PDT is first order and then merges with the first-order wetting step (if it exists). Indeed, it is possible that upon the increase in solvent concentration, the prewetting step and the (smooth) PDT are initially disconnected and then merge to a combined step-like transition. An example of where such merging event is found is illustrated by the unnumbered blue dot in Figure 6a.

The diagrams in Figure 6 may be interpreted as wetting phase diagrams. Below the black line, the system is wet by the PEG-rich phase. Above the black line, the system is either in the partially wet state or in the dry state (c.q. the surface is wet by the dextran-rich phase). As the interfacial tension in these systems is very low, and the drying transition is not very far from the WT (for an example, we refer to case 4 discussed below). In other words, the window for partial wetting is very small (especially close to the bulk critical point). All of this is in line with the Cahn conjecture.

In passing, we can elaborate why a drying transition line can exist above the WT line. Polymer adsorption from a solvent is an exchange process. That is, as soon as a polymer segment adsorbs, a solvent molecule must be removed from the surface. In good solvent conditions, the adsorption energy is solely given by the interaction parameter of the polymer segment with the surface. However, in marginal or theta-solvent conditions, the effective adsorption energy also becomes a function of the solvent concentration. In our systems, the dextran intrinsically had a lower affinity for the surface while they were in the worse solvent conditions. Then, the effective surface affinity increases with increasing solvent concentration and this increase is stronger for dextran than for PEG, explaining why the system can switch from a higher surface preference for PEG to a higher preference for dextran.

### 3.3 Major Driving Force (Open Binodal)

ATPS driven by a solvent quality mismatch (minor driving force), as considered in the previous paragraph are exceptional. More likely, the segregative phase behavior is driven by a repulsion between the two types of polymer, parametrized by χ_*PD*_. This is why we call this scenario the major driving force. As illustrated above, the corresponding phase diagram is ’open’. For equal degrees of polymerization, and sufficiently large *N*≡*N*_*P*_ = *N*_*D*_, even a modest repulsion between the two types of segments suffices to dominate over the minor driving force (cf. case 2). For simplicity, we will take athermal solvent conditions χ_*PS*_ = χ_*DS*_ = 0. Under these conditions, the solvent concentration is the same in both phases and we can refer to the bulk volume fraction of solvent φ_*S*_^*b*^ without the need to specify which of the two bulk phases it refers to. In this symmetric case, it is easy to see that when the two components have the same affinity for the surface, that is, when Δχ_*W*_≡χ_*WD*_–χ_*WP*_ = 0, we have the ’neutral’ condition and the system is in the partially wet state as the contact angle must be θ = 90° for all values of the control parameter Δφ_*S*_ ≡ φ_*S*_^cr^-φ_*S*_^*b*^. For *N* = 100, the critical volume fraction of solvent is φ_*S*_^cr^ = 0.9.

In the symmetric system described above (cf. case 3) the system is in the two-phase state when φ_*S*_^*b*^<φ_*S*_^cr^. We take χ_*WP*_ < χ_*DP*_, again because we assume that the particles have a higher affinity for PEG than for dextran. Hence, our interest is in the case that Δχ_*W*_ > 0. When this value is sufficiently large we find that the contact angle θ = 0 for solvent volume fractions typically used in ATPS. To find partial wetting one has to focus on relatively small values of Δχ_*W*_ and/or to small values of Δφ_*S*_, that is, we have to use high amounts of solvent. In Figure 7a we present how the contact angle goes from a finite value to zero upon a change of the control parameter to zero for fixed χ_*DW*_ = – 4 and various values of Δχ_*W*_, while in Figure 7b the χ_*WD*_ was varied and fixed Δχ_*W*_ = 0.01. In these graphs the θ = 90° corresponds to the – *S* = 1–cos θ = 1, whereas the wetting transition corresponds to 1–cos θ = 0.

**Figure 7:**
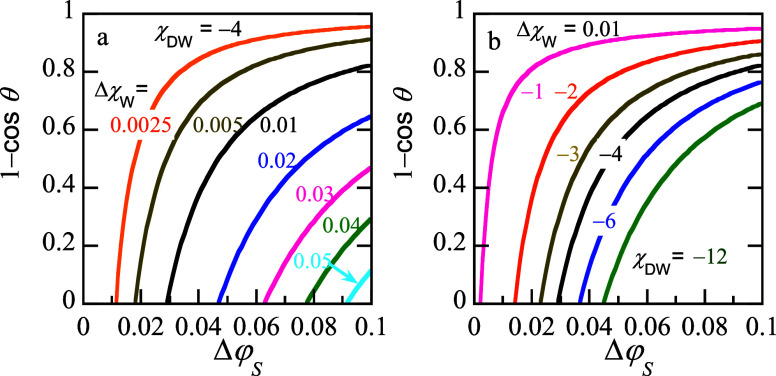
–*S* = 1–cos θ = γ_thick_–γ_thin_ as a function of the shifted volume fraction of solvent Δφ_*S*_ ≡ φ_*S*_^cr^-φ_*S*_^*b*^ (a) χ_*DW*_ = – 4 and various values of Δχ_*W*_ = φ_*DW*_ – φ_*PW*_ as indicated. (b) Δχ_*W*_ = 0.01 and values for χ_*DW*_ are indicated. Other parameters (see parameter case 3): χ_*DP*_ = 0.2, *N*≡*N*_*P*_ = *N*_*D*_ = 100 (strong driving force), χ_*PS*_ = χ_*DS*_ = 0 (no minor driving force).

Inspection of the contact angle predictions of Figure 7 reveals that all the results are fully in line with the Cahn argument: for cases that θ ≠ 0 (partial wetting) we find a wetting transition θ → 0 (WT), when the control parameter is on the way to Δφ_*S*_ → 0. In all graphs, the slope of ∂(1–cos θ)/∂Δφ_*S*_ changes abruptly at the wetting transition, that is, when 1–cos θ = 0. This implies that the wetting transition is first-order. Again, this result is unusual, as in classical wetting theory it is found that when the wetting transition occurs near the bulk critical point, it becomes second order.

When both polymers have a significant affinity for the surface χ_*DW*_ = – 4 (strong adsorption), one approaches neutral conditions when Δχ_*W*_ → 0 (panel a). For given Δχ_*W*_ one will go toward the neutral condition when both polymers have progressively less affinity for the surface. Indeed χ_*WD*_ ≈ – 1 is close to the critical adsorption energy and when Δχ_*W*_ is small also χ_*PW*_ ≈ – 1 and PEG is close to the critical adsorption strengths as well. When both polymers avoid contact with the surface, the solvent starts to become the interfacial species and the system is close to the neutral value (panel b). Near the neutral value the contact angle remains high (close to 90°) for a wide range of Δφ_*S*_ values until very close to Δφ_*S*_ = 0 where the contact angle drops quickly to zero. This very strong drop is rationalized by realizing that in these conditions the interfacial tension between the PEG-rich and dextran-rich phases is very low (cf. Figure 2b). When the WT occurs at rather high values of the control parameter Δφ_*S*_, the variation of the contact angle changes less abruptly with Δφ_*S*_.

It is instructive to discuss the adsorption–displacement transition also when there is a significant repulsion between PEG- and dextran chains, χ_*PD*_ = 0.2, so that the system can be in the two-phase state when the volume fraction of solvent is less than 0.9 (that is, when *N* = 100). We have seen above that wetting transitions in these systems tend to be of the first-order type. This implies that there is a prewetting step in the isotherm when the system is in the complete wet state. It was found that the displacement of adsorbed dextran by PEG happens together with the prewetting transition (or vice versa) (not shown). Apparently, when the major driving force sets the bulk phase behavior, it is likely to have an energy barrier that has to be overcome to switch from an excess of dextran to an excess PEG chains (and a depletion of dextran). For similar parameter settings, we may consider the displacement transition for critical, or even supercritical conditions, that is, when Δφ_*S*_ < 0.

For the critical volume fraction of solvent φ_*S*_^*b*^ = 0.9, we present in Figure 8a adsorption isotherms of PEG (solid lines) and the displacement of dextran as a function of the bulk volume fraction of PEG in the system for various values of χ_*PW*_ < χ_*DW*_ = – 4.0. Inspection of this graph proves that the displacement transitions are strongly first-order even when Δχ_*W*_ is small. This is seen by the presence of a van der Waals loop in the isotherm. Importantly, the results show that the loop has no tendency to vanish in the limit of low values of Δχ_*W*_ and thus we should expect that loops in the isotherm remain present for conditions used in the wetting study (cf. Figure 7).

**Figure 8:**
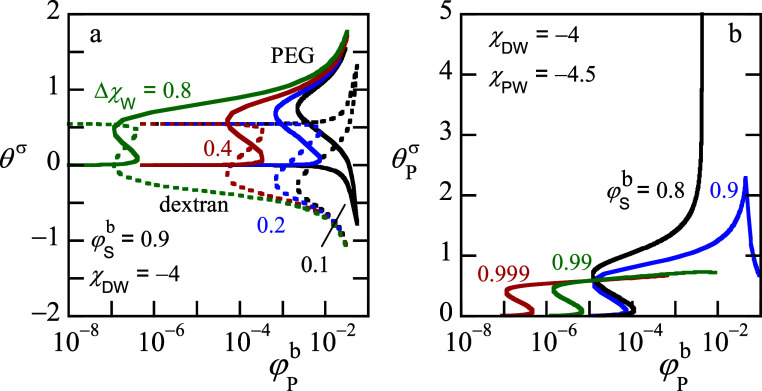
(a) Excess adsorbed amount θ^σ^ of PEG (solid lines) and dextran (dotted lines) as a function the volume fraction of PEG in the bulk phase φ_*P*_^*b*^ in lin-log scale. Δχ_*W*_-values are indicated, while χ_*DW*_ = – 4 and φ_*S*_^*b*^ = 0.9 (critical value). (b) Excess adsorbed amount of PEG θ_P_^σ^ as a function of the bulk volume fraction of PEG φ_*P*_^*b*^ in lin-log scale for various values of the volume fraction of solvent φ_*S*_^*b*^ as indicated. χ_*DW*_ = – 4 and χ_*PW*_ = – 4.5 (hence Δχ_*W*_ = 0.5). The adsorbed amount of dextran is not plotted but roughly mirror-images the adsorbed amount of PEG (as in panel a). Other parameters as in case 3: *N* = 100, χ_*PD*_ = 0.2 and χ_*PS*_ = χ_*DS*_ = 0.

In Figure 8b we present adsorption isotherms for PEG for various values of φ_*S*_^*b*^ while the interaction parameters with the surface remain fixed to χ_*DW*_ = – 4 and χ_*PW*_ = – 4.5. Again, the isotherm for φ_*S*_^*b*^ = 0.9 (blue line) corresponds to the critical value and is similar to the curves plotted in panel a). Indeed, when the θ_P_^σ^ increases jump-like that of dextran decreases (and even becomes negative) as in panel a) but we did not plot the adsorbed amounts of dextran in this figure. When φ_*S*_^*b*^<φ_*S*_^cr^ = 0.9 we see the classical isotherm for a system that is wet by the PEG-rich phase. The isotherm continuously increases and diverges at the bulk binodal. The step in this isotherm is classically referred to as the prewetting step and here coincides with the displacement transition. The isotherm for the critical value (blue curve) has a cusp at φ_*P*_^*b*^ = 0.05 and drops to lower value at higher bulk volume fractions. In (panel a) this drop was not shown for presentation purposes. Even when the bulk volume fraction of solvent is much higher than the critical value, the displacement transition remains first order and shifts progressively to lower values of the PEG bulk volume fraction. This explains why the wetting transitions were found to be first order. In particular, it must be expected that even when the wetting transition takes place at the critical value (that is when the system is exactly in the neutral case), the wetting transition remains first-order. At the same time, when the control parameter is further increased to even better wetting conditions, one will typically find a surface critical point, wherefore the step in the isotherm is lost. However, for ATPS, the prewetting step can also occur very far from the saturation value. Of course, this again is attributed to the displacement transition, which for long enough chains and sufficiently strong repulsion between the chains, is first order and this displacement transition can occur very far from bulk saturation (hence the prewetting step is found exceptionally far from the bulk saturation value).

### 3.4 Experimental Case Study

In Figure 9 we present predictions for the wetting phase diagram for the experimental system in units Δχ_*W*_ vs φ_*S*_^*b*^ in the dextran rich phase, or Δχ_*W*_ versus the affinity of dextran for the surface χ_*DW*_. In (panel a) the adsorption strength for the dextran segments is fixed to φ_*WD*_ = – 4 (strong adsorption), whereas in (panel b) the volume fraction of solvent in the dextran-rich phase is fixed φ_*S*_^(D)^ = 0.8. In these phase diagrams one can find the regions for parameters corresponding to completely wet-, partially wet and completely dry systems. In (panel a) it is noticed that the wetting transition line and the drying line come together at the bulk critical point (vertical dotted line in panel a) as anticipated above and in line with the Cahn conjecture. Indeed, in this (near critical) region, the window of partial wetting is very narrow. We may estimate the neutral condition (contact angle 90°) to be approximately halfway in between the wetting and drying lines. It is noticed that the neutral condition is not at a given Δχ_*W*_ because the solvent quality for dextran is taken to be worse than that for PEG. Under such solvency conditions, the effective adsorption strength is not only given by χ_*PW*_ and χ_*DW*_, but is also solvent concentration- and solvent quality dependent (χ_SD_ = 0.48 and χ_SP_ = 0.44). The neutral conditions are also affected by the chain length difference (*N*_*P*_ = 100 and *N*_*D*_ = 300). The Δχ_*W*_(neutral) increases slightly with increasing volume fraction of solvent φ_*S*_ and is approximately 0.1 at φ_*S*_^(D)^ = 0.7 to close to 0.2 near φ_*S*_^cr^. This value decreases to approximately 0 when χ_*WD*_ = – 1 (near critical adsorption strengths).

**Figure 9:**
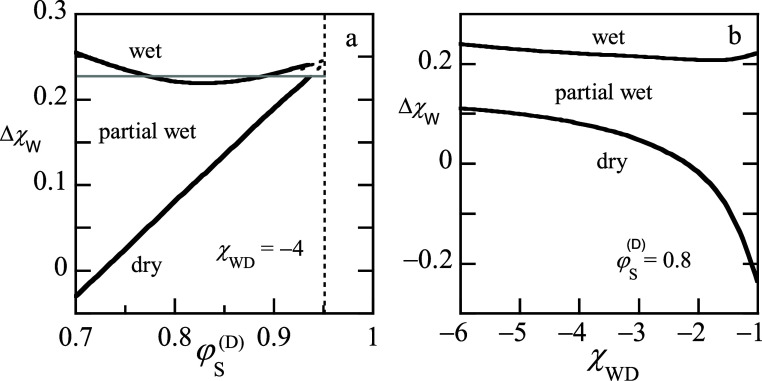
(a) Wetting phase diagram in units Δχ_*W*_ = χ_*DW*_–χ_*PW*_ versus the volume fraction of solvent in the dextran-rich phase φ_*S*_^(D)^ while the adsorption strength for dextran units is fixed to χ_*DW*_ = – 4. The vertical dotted line is at the critical volume faction of solvent. The horizontal gray line is discussed in the text. (b) Wetting phase diagram in units χ_*W*_ = χ_*DW*_–χ_*PW*_ versus the adsorption strength of dextran units χ_*DW*_ at fixed volume fraction of solvent φ_*S*_^(D)^ = 0.8. The top curves are the wetting transition lines; the bottom curve are the drying transition lines. These lines demarcate the parameter regions for wet, partial wet and dry systems. Other parameters, see case 4.

Inspection of Figure 9a reveals that the wetting transition line goes through a narrow minimum. This has interesting consequences. As indicated by the gray horizontal line, it is possible by changing the amount of solvent (in the dextran rich phase) from a low value toward critical, to observe three consecutive wetting transitions. (i) From partial to complete wetting (just below φ_*S*_ = 0.8), (ii) from complete to partial wetting (just above φ_*S*_ = 0.85), (ii) from partial wetting to dry (just before the critical solvent value). In panel b) the concentration of solvent (water) is fixed to a value of 0.8 (a typical water content wherefore experiments are feasible). Upon a change of the adsorption strength of dextran one can also have a re-entrant wetting behavior, where partial wetting at strong adsorption, complete wetting at intermediate adsorption strengths, back to partial wetting near the critical adsorption strength. The drying line is monotonic and no re-entrance behavior is expected in this part of the phase diagrams.

From an experimental point of view we must conclude once again that the window of partial wetting is narrow. Very likely, in experiments the surface affinities χ_*PW*_ and χ_*DW*_ do differ more than 0.2 *k*_*B*_ *T* and then the systems are in the completely wet state for all values of the control parameter φ_*S*_. Again, this is in line with our experimental observation that silica and polystyrene particles invariably partition in the PEG-rich phase.

The rather narrow window for partial wetting and thus the wide windows for completely wet cf. completely dry states are directly linked to the rather low values of the interfacial tension (cf. Figure 3b) between the PEG-rich and dextran-rich phases and the fact that this interfacial tension occurs in the denominator of Young’s law (cf. 1. Another way to rationalize that the partial wetting condition is likely to exist only far from the critical point is to consider the interfacial width (cf. Figure 3b). Particles are only expected to have an affinity for the interface when the particle size is large compared to the width of the interface. In the other limit, that is, when they are small compared to the width of the interface, they no longer can locate the interface and hence cannot adsorb onto it. Again, taking the solvent amount as the control parameter, it is clear that the interfacial width diverges upon the approach of the solvent volume fraction toward the critical point, hence they will loose interfacial activity. Pickering stabilization of interfaces in aqueous two-phase systems is therefore expected only at relatively low water concentrations and for systems with carefully tuned interaction parameters.

We have argued above that the wetting transition remains first-order even when it occurs very close to the bulk critical point and that this is not the classical result. Classically, one expects that the wetting transition becomes second order in this limit. In order to reconcile a first-order displacement transition in tandem with a second order wetting transition, one needs an isotherm that crossed the binodal twice (once for the first-order displacement transition and a second time for the second order wetting transition) before the isotherm diverges at the binodal (where it merges coming from supersaturation concentrations). Such type of isotherms are known to exist for system with surface interactions on two different length scales.^37,38^ More specifically, there exists the pseudopartial wetting scenario: the short-ranged interactions call for complete wetting (loop in the isotherm), while longer-ranged interactions prevent the smooth divergence of the adsorbed amount of the wetting component and the isotherm crosses the binodal for the second time. A polymer brush in a binary liquid with a solubility gap may provide such scenario. The solvation of the polymer brush by the wetting liquid is first-order, while the smooth detachment of the liquid–liquid interface from the brush is prevented by the adsorption of the terminal ends of the brush-chains at the liquid–liquid interface. Indeed, a finite adsorption leads to a barrier that prevents the smooth growth of the wetting layer and hence the second crossing of the binodal manifests. In our system such “barrier” that prevents the growth of the wetting film thickness is not expected to exist. It is known that the interface between the PEG-rich and dextran-rich phases is depleted by both polymers, while the low molecular weight solvent accumulates at it. The depletion of PEG chains at the interface simply cannot invoke a “barrier” (this is possible for adsorbing chains only). Hence, we must expect that after the (jump-like) adsorption displacement transition occurred there can only be a continuous growth of the wetting film upon the approach to the binodal (and it merges with the binodal from subsaturation concentrations) and there is no second crossing of the binodal. Hence, the pseudopartial wetting scenario is not possible for ATPS and the wetting transition is simply first-order.

## 4 Conclusions

Using a numerical self-consistent field approach, we have analyzed both the adsorption and wetting scenario in three-component systems, consisting of two types of polymer in a common solvent. The dextran PEG and water system is a typical example. There may be two segregative driving forces that give rise to aqueous two-phase systems. When there is only a solvent quality difference, it is possible to find a closed-loop binodal (rare case). However, when there exists a reasonable repulsion between polymer segments χ_*DP*_, one finds open binodals (common scenario).

We studied wetting scenarios using adsorption isotherms of a minority (component) forming a minority phase in the system, where the concentration of the majority polymeric component is fixed. Upon increasing concentration, the adsorption layer must switch in the adsorbing species from the majority to the minority one. Hence, along the adsorption isotherm a displacement transition takes place. Only in very ideal situations, when there are no unfavorable interpolymeric interactions and when the solvent quality mismatch is weak (or amount of solvent is low), the displacement transition is smooth. In all other cases it is first order (the adsorption isotherm features a van de Waals loop).

When parameters are such that the system has a solubility gap, i.e. macroscopically there is an aqueous two-phase system, and this system is studied near a solid interface, wetting scenarios can be studied. Classical wetting theory allows wetting transitions from partial to complete wetting (or complete drying) to be first- or second-order. The latter is typically only found near bulk critical points. However, in our system, the displacement transition merges with the prewetting transition and this causes the wetting transition to be first order, even up to the situation that the wetting transition occurs at the bulk critical point. Because the prewetting step in the isotherm frequently merges with the adsorption displacement transition, it shows nonclassical behavior from a wetting perspective. In particular the first-order displacement transition may also occur while the bulk is supercritical in a one-phase state. Then we should classify the displacement transition as a pure surface phase transition.^19^

We have systematically studied the windows for partial wetting in various three-component systems. Invariably, we found this window to be very narrow. Also, for our experimental system we could find only small regions of parameter space for which the system is partially wet. This explains why in aqueous two-phase systems it is hard to stabilize emulsions by interfacial particles (known as Pickering emulsions). The highest probability to find particles at interfaces is by going to the limit of low solvent volume fractions (far from critical), but even then the window is narrow. In the experimental case, the system is far from symmetric: The dextran chains are much longer than the PEG chains, and water is a poorer solvent for dextran than for PEG. In such systems, at least in principle, it is possible to find, upon an increase of solvent (water) in the system, three consecutive wetting transitions: partially wet → wet → partially wet → dry. However, experimentally, we found a more boring result, i.e., both silica particles and polystyrenesulfonate ones reside in the PEG-rich phase for a wide range of solvent contents in the system. These observations suggest that the strength of adsorption for silica or polystyrene particles of PEG and dextran differs more than a few tenths of *k*_*B*_ *T*.

In a forthcoming paper, we will apply our wetting results for the experimental system and extend the analysis to capillary condensation and eventually to capillary suspensions.
